# Cangpu Oral Liquid as a Possible Alternative to Antibiotics for the Control of Undifferentiated Calf Diarrhea

**DOI:** 10.3389/fvets.2022.879857

**Published:** 2022-04-29

**Authors:** Shengyi Wang, Dongan Cui, Yanan Lv, Zuoting Yan, Jiyu Zhang

**Affiliations:** Key Laboratory of Veterinary Pharmaceutical Development of Ministry of Agriculture and Rural Affairs, Lanzhou Institute of Husbandry and Pharmaceutical Sciences of Chinese Academy of Agricultural Science, Lanzhou, China

**Keywords:** calf diarrhea, cangpu oral liquid, Chinese herbal medicine, randomized controlled trial, antibiotic alternative

## Abstract

Antibiotics are essential in the prevention of calf diarrhea epidemics. As more antibiotics become ineffective due to drug-resistant bacteria, attention must be directed toward alternative treatments for calf diarrhea. Natural antibiotic alternatives, such as Chinese herbal medicine, have become a research hotspot in the clinical treatment of diseases such as calf diarrhea due to their characteristics of fewer side effects, low cost, little residue, and no drug resistance. The Cangpu Oral Liquid (CP) was modified from a traditional herbal formula that had been widely used in ancient China to treat gastrointestinal diseases in animals. In order to evaluate the treatment effect of CP on neonatal calf diarrhea, a randomized controlled field trial was performed. Two hundred and forty-six diarrheal Holstein calves of 2–15 days old were selected and randomly divided into two treatment groups receiving either apramycin or CP. 101 out of 123 calves recovered from diarrhea in the CP group, whereas 77 out of 123 calves showed recovery after antibiotic therapy. There were no differences in initial weight between both groups, while the final weight was significantly different (*P* = 0.892, *P* = 0.025, respectively). The mean average daily gain (ADG) of calves (211.45 gram/day) in the CP group was significantly higher compared to the antibiotic group (164.56 gram/day) (*P* = 0.001). The CP group also showed a shorter recovery time from diarrhea (3.90 days vs. 6.62 days, *P* = 0.001). The current results indicate that the CP has a beneficial clinical effect on the treatment of diarrhea in neonatal calves and is an effective alternative treatment option.

## Introduction

The yearly survival calf ratio always measures the proliferation and development of cattle industries (1). However, the earlier onset of diarrhea in neonatal calf has been detrimental to the industry and affected their products worldwide. Neonatal calf diarrhea is a serious disease with a complex etiopathogenesis, high morbidity, and mortality, causing huge economic loss in beef or dairy farms (2). According to the literature, diarrhea or other digestive problem almost accounted for 14% of death in calf under 3 weeks and 23% of deaths in calf over 3 weeks (3). Environmental stress, management style, nutritional state, immune status, and enteric pathogens are major risk factors of calf diarrhea (4). The mostly reported enteric pathogens were bovine rotavirus (BRV), bovine coronavirus (BCoV), *Escherichia (E.) coli* and *Cryptosporidium (C.) parvum* (5). Other infectious agent such as bovine viral diarrhea virus (BVDV), *Salmonella (S.) enteric, Clostridium (C.) perfringens*also were detected in fecal samples (6). Harsh weather conditions such as uncomfortable temperatures, rain, wind, and poor hygiene conditions could increase the susceptibility of calves to diarrhea (7). The quality and quantity of colostrum are also associated with the occurrence of calf diarrhea (8).

Antibiotics is a major way to prevent calf diarrhea outbreaks and have been used without being guided most of the time (9). Due to the widespread use of antimicrobials in animal husbandry, the possibility of cross-resistance between human and animal pathogens has aroused much public concern (10). Thus, the use of antibiotics is in a dilemma situation due to the occurrence of antimicrobial resistance. Antibiotic susceptibility testing is the main strategy to guide clinical antibiotic use (11). However, fecal bacterial species do not accurately mirror the intestinal bacterial species, and the breakpoints of susceptibility test results have not been reproducible (12). Therefore, it is urgent to develop alternative strategies to address this disease in calves. Some alternative treatments for diarrhea have been studied recently, including zinc, crofelemer, lactoferrin, cinnamaldehyde, and bacteriophages targeting enteropathogens (13–17). Ethnoveterinary or Chinese herbal medicine has become a research hotspot in the clinical treatment of diseases such as calf diarrhea due to its characteristics of fewer side effects, low cost, almost no residue, and no drug resistance (18–20).

Ethnoveterinary medicines play a pivotal role in animal health care worldwide, and medicinal plants are always used to treat calf diarrhea (21–25). Traditional Chinese Veterinary Medicine (TCVM), which has also been used to treat all kinds of animal diseases for centuries, may also be an ideal option for an antibiotic alternative for neonatal diarrhea. Cangpu oral liquid (CP) was a modified preparation from the classic TCVM formula of pingwei powder, which was first documented in the Yuan-heng-liao-ma-ji and widely employed to treat gastrointestinal diseases in animals. According to TCVM theory, CP was used to treat diarrhea with the syndrome of cold-dampness encumbering the spleen, and the function of which was warming the interior to dissipate cold, astringing the intestines and check diarrhea. A preliminary experiment indicated that the CP had a good therapeutic effect on calf diarrhea. This study evaluates the efficacy and potential as an antibiotic alternative of the CP in field cases of undifferentiated calf diarrhea.

## Materials and Methods

### CP Preparations

The formula of CP is composed of five herbs (80 g of *Atractylodis Rhizoma*, 60 g of *Magnoliae Officinalis Cortex*, 50 g of *Coptidis Rhizoma*, 50 g of *Psoraleae Fructus*, 60 g of *Mume Fructus*), and all these herbs were purchased from Hui Ren Tang Chinese Medicine Co., Ltd. (Lanzhou, China). The quality of the herbs meets the Veterinary Pharmacopeia of the People's Republic of China (Chinese Veterinary Pharmacopoeia Commission, 2015). After pre-processing with washing, drying, and chopping, the mixed herbs were boiled three times for 1.5 h each time with 10 times purified water of the total herbal weight. The extract was collected and concentrated to 300 mL by boiling, resulting in a final concentration of 1.0 g crude herb/mL for the experiment.

### Study Population

All experiment procedures were approved by the Ethics Committee of Lanzhou Institute of Husbandry and Pharmaceutical Sciences of the Chinese Academy of Agricultural Sciences (Approval No. LZMY 20021-0015). The clinical trial was conducted from May to July 2021 in the Reproduction and Breeding Demonstration Center of Chinese Holstein Dairy Cow of Gansu Province (36°19′N, 103°18′E), where reared approximately 5000 Holstein calves, 2000 of which were of pre-weaning age. Within 4h after birth, all calves were fed 4 L of colostrum with optimum quality by suckling or via the oro-esophageal feeder. The colostrum was collected within 1 to 2 h after calving and all calves were moved to individual outdoor hutches within 24 h of birth.

The study was devised to provide sufficient statistical power to achieve a difference in diarrhea resolution between groups. Based on the preliminary results, a 63% cure rate in the antibiotic group and a 80% cure rate in the CP group, 110 animals per group would provide sufficient power (0.80) to detect a significant difference at α = 0.05. Allowing for a 10% loss to follow-up, more than 244 calves should be included and assigned to the two groups in this trial.

### Enrollment/Exclusion Criteria

All calves were visually observed each morning by one of the co-authors during the first 15 days of life. The fecal consistency scores and dehydration scores were recorded according to previously described methods. Briefly, fecal scores were graded as follows: 1 (formed), 2 (semi-formed pasty), 3 (loose but stays on top of the bedding), or 4 (watery diarrhea that sifts through the bedding). Dehydration scores were recorded as 1 (no signs of dehydration), 2 (mild depression, skin tent in the neck region of 2–6 s, recessed eyes), 3 (skin tent >6 s, very recessed eyes, and the calf disinclined or reluctant to get up), or 4 (calf will not stand, skin does not flatten when tented) (26). Pre-weaning calves were enrolled if they developed diarrhea (fecal scores of 3 or 4) for the first time. Calves were rejected from the study if they had serious dehydration (dehydration score >2) or symptoms of disease other than diarrhea, such as an umbilical abscess, pneumonia, or meningitis, or if they needed immediate antibiotic therapy.

### Study Design and Treatment Protocol

This study was devised as a randomized controlled trial. After enrollment, calves were sequentially randomized to one of two treatment groups in bulks that ranged from 2 to 15 calves according to the number of calves qualified at each enrollment day by using random-number generated by Microsoft Excel (Microsoft Crop., Redmond, WA). To limit the awareness of the investigator to assessment assignment, treatment allocation, administration was performed by one co-author and the data recording and treatment assessment were carried out by another co-author who was blinded for the treatment until the completion of the trial. Calves received twice daily an oral dose of 100 mL of CP or twice daily an oral dose of 12 mg/kg of apramycin for four consecutive days. When calves had a fecal score of 1 or 2, a dehydration score of 1 in 2 consecutive days, they were regard as clinically cured.

### Data Collection

Upon enrollment, the baseline characteristics of each calf were recorded including age, fecal scores, and dehydration scores. Calves were weighed respectively using a digital scale at enrollment and exit (day 10). Scale weights were not available for the first 10d of the study, and these calves were excluded from the analysis portion. Each calf received a complete physical examination each day and a maximum of 10 d following enrollment, or until calves had normal clinical parameters for two consecutive days. The fecal scores, dehydration scores, suck reflex, and other clinical abnormalities were recorded during the physical examination Disease duration was described as the number of days between enrollment and the first day when the calf was defined as clinically cured. Calves were culled if farm staff regarded that the severity of the disease needed other treatment.

### Statistical Analysis

Means, proportions, and standard errors were displayed for baseline characteristics of calf diarrhea cases. General Linear Model (GLM) was used to testfor differences between treatment groups in weight at enrollment, final weight, and average daily gain (ADG). The differences in the proportion of calves with a fecal score of 4 or a dehydration score of 2 were tested by a chi-squared test. A nonparametric ANOVA (Kruskal-Wallis test) was used to compare age at enrollment between groups. Kaplan–Meier analysis was used to determine differences between groups in disease duration. Calves were right-censored if they died, culled, or had a fecal score >2 at exit (day 10). A Cox Proportional Hazard (PH) regression analysis estimated the hazard ratio for treatment. The proportional hazard assumption that clinical cure hazard is independent of time was assessed using each treatment group's log-minus-log (LML) survival plots. Statistical significance of the model parameters was tested using the Wald Chi-Square test.

## Results

### Enrollment

**Figure 1:**
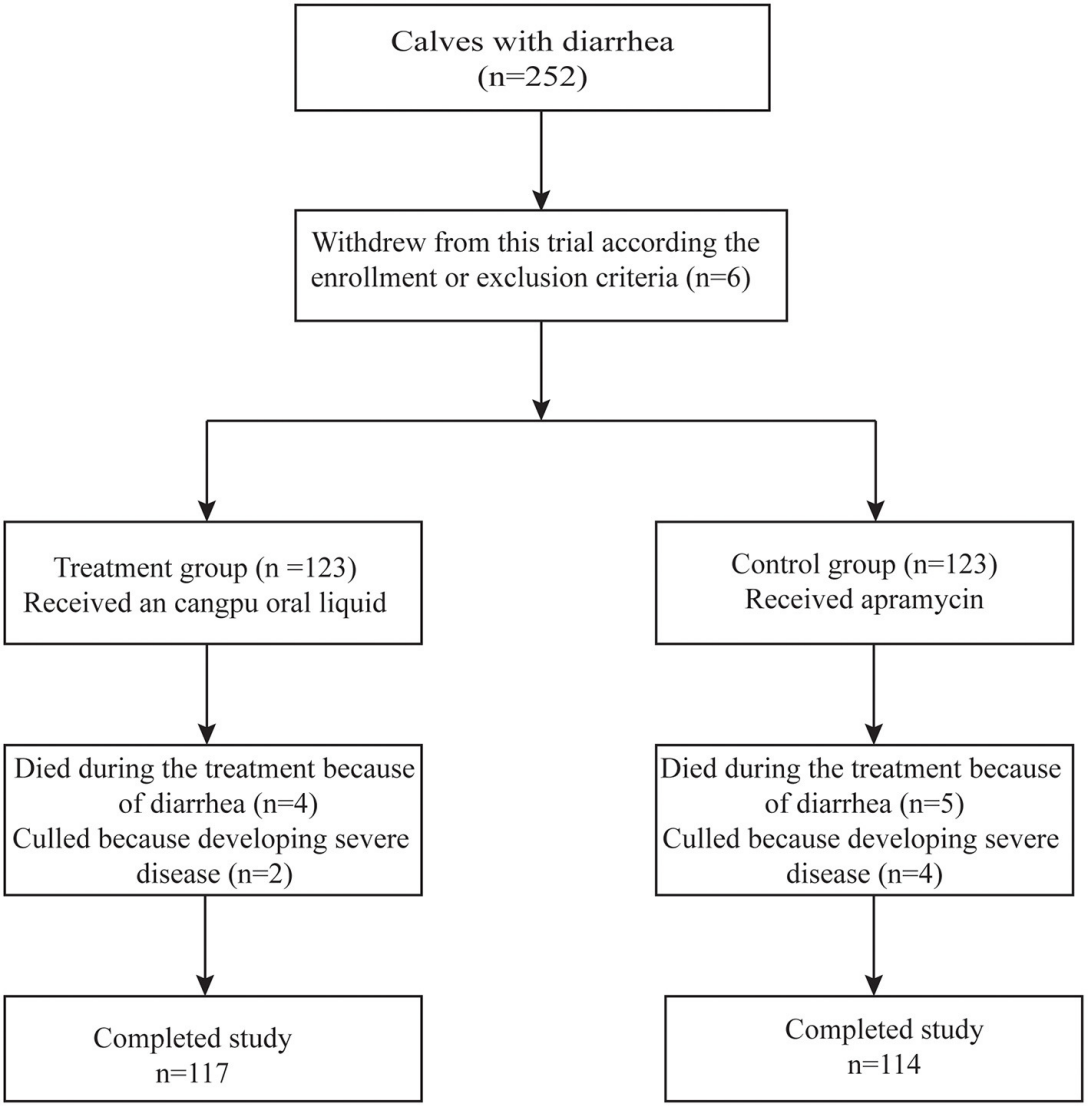
Flow diagram showing the calves affected with diarrhea, randomization of calf with diarrhea, reasons for excluding calf from analyses, and completed study for statistical analysis.

Two hundred and fifty two (252) calves were enrolled according to the inclusion criteria (126 calves in each group). Six calves were excluded from the trial including three calves in the CP group (two died immediately after enrollment and another one was enrolled with pneumonia) and three calves in the control group (all were enrolled with serious systemic disease and were unable to drink milk). All of the 246 calves included in this study, 15 died or were culled in the following 10 d: four died and two were culled in the CP group while five died and four were culled in the control group. Figure 1 shows the disposition of the enrolled calves.

### Baseline Comparison

The result of baseline comparisons showed that there were no significant differences in age (*P* = 0.192), initial weight (*P* = 0.892), percent with an initial fecal score of 4 (*P* = 0.779), and percent with an initial dehydration score of 2 (*P* = 0.755) between the two treatment groups at enrollment (Table 1).

**Table 1:** The characteristics of calves in a randomized clinical trial on the effect of CP and apramycin on the treatment of calves for diarrhea.

**Item**	**CP**	**apramycin**	**SE**	***P*-value**
Mean age (*d*)	5.80	6.20	0.310	0.192
Calves with fecal score = 4[% (*n*)]	46.3	41.5	/	0.779
Calves with dehydration score = 2[% (*n*)]	61.8	59.3	/	0.755
Day 1 weight (kg)	43.14	43.17	0.177	0.892
Day 10 weight (kg)	45.26	44.81	0.198	0.025
10-d ADG (g/d)	211.45	164.56	5.23	0.001

### Weight Measurements

**Figure 2:**
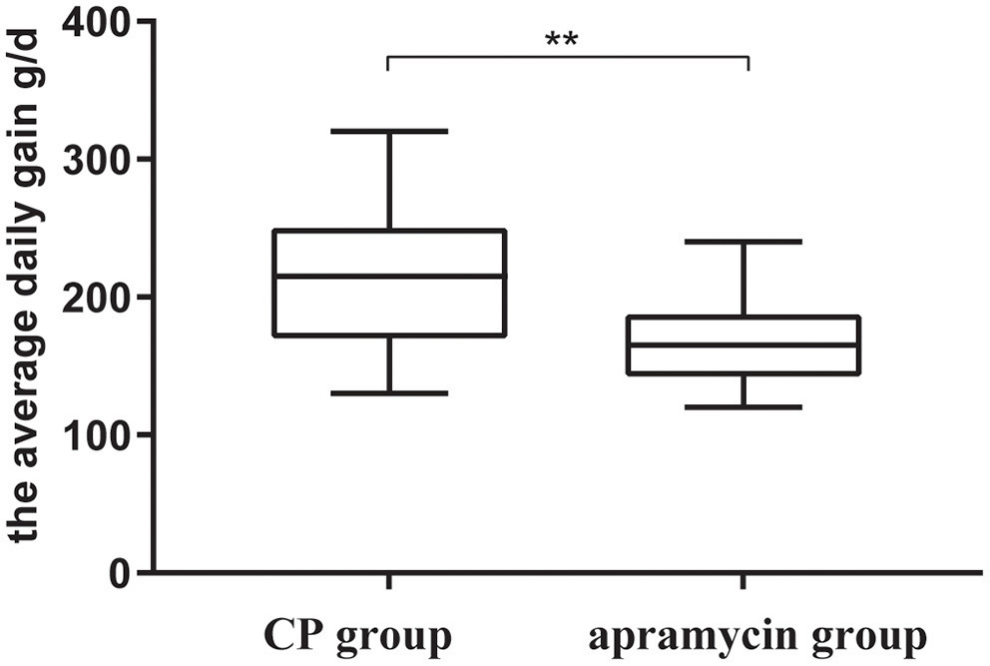
The average daily gain of the calves at enrollment and exit received a twice daily oral dose of 100 ml per time of CP or a twice daily oral dose of 12 mg/kg per time of apramycin, **p < 0.01.

Body weight at exit was weighed for 117 and 114 calves in the CP and control treatment groups, respectively (Table 1). The final body weight of the CP group was significantly better than that of the control group (*P* = 0.002), and the mean ADG of calves in the CP group (211.45 g/d) was significantly (*P* = 0.001) better than the control group (164.56 g/d) (Figure 2).

### Clinical Cure

**Figure 3:**
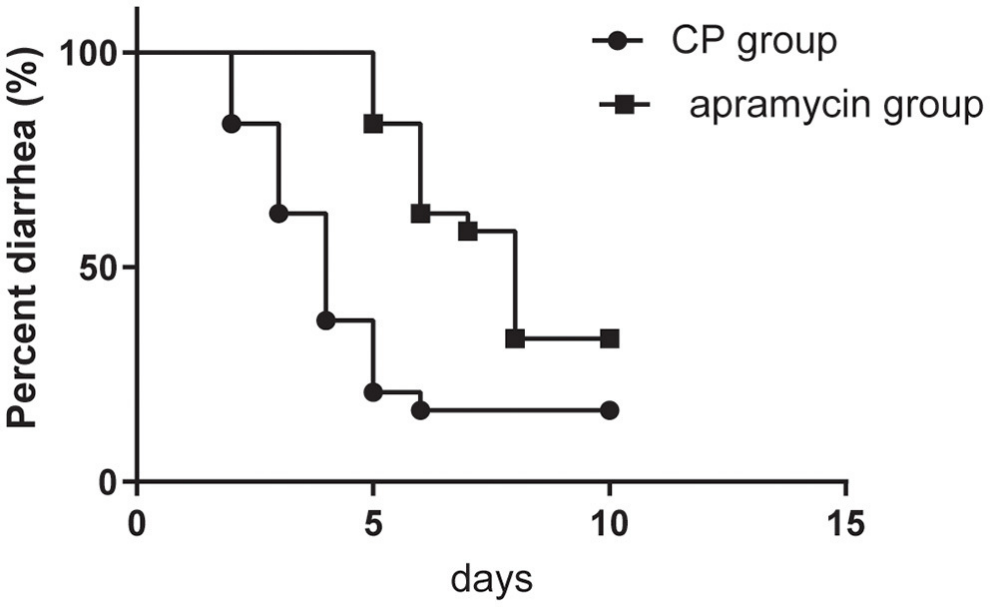
Kaplan-Meier survival curve describing the time needed for recovery after the onset of diarrhea at the 123 diarrheal calves in the group of calves receiving CP and at the 123 diarrheic calves receiving apramycin treatment.

In the present study, 101 of 123 calves recovered from diarrhea following the treatment with CP, whereas 77 of 123 calves showed recovery after apramycin therapy. The mean days to recovery from diarrhea of calves between the two group were significant differences (*P* = 0.001) (Figure 3). The CP group showed a shorter recovery time from diarrhea (3.90 days vs.6.62 days, *P* = 0.001).

Based on the Cox Proportional Hazard (PH) regression analysis, the interaction between the two groups and initial fecal score, age, and weight was not significant (*P* > 0.05). Cox Proportional Hazard (PH) regression analysis also suggests that the initial fecal score of 3 or 4 had no significant interaction with the calf recovery from diarrhea (HR=1.116). Calves treated with CP had 2.923 times higher of a clinical cure at exitcompared to calves in the control group (*P* = 0.002).

## Discussion

Randomized controlled trials are necessary to evaluate the effectiveness of alternatives to conventional therapies with antibiotics (15). Due to the misuse of antibiotics in the livestock industry, the possible cross-resistance between human and animal pathogens has attracted wide public concern. Antimicrobials have been reported to alter intestinal flora and function by depletion of the lumen down to the layer of the organism (27). The adoption of rapid antibiotic susceptibility testing is often used to guide clinical antibiotic usage (28). However, reports indicated that fecal bacterial culture and antimicrobial susceptibility testing were not recommended in calves with diarrhea because fecal bacterial populations do not accurately reflect small intestinal or blood bacterial populations (29). In addition, the breakpoints for susceptibility test results have not been validated (12). Therefore, alternative treatments to antimicrobial have been investigated for many years. The CP used in this study was modified from a traditional herbal formula that had been widely used in ancient China. Medical literature suggests that apramycin significantly decreased the mortality rate in calves treated at 20 mg/kg PO q24h for 5 days (mortality 9%, *P* < 0.01) or 40 mg/kg PO q24h (mortality 6%, *P* < 0.01) when compared with untreated controls (mortality 30%) (11). In this trial, calves treated with CP had a higher clinical cure at exit compared to the controls and the CP could improve short-term outcomes of the diarrheal calves in weight gain and disease duration. It suggested that the CP had a beneficial effect on calf diarrhea as a treatment option under field conditions.

Calf diarrhea severely affects calf growth and subsequent production performance. The loss of water content and disease duration could aggravate the loss of water of the calf suffering from diarrhea to some extent. Hartmann and Reder described that calves suffering from watery diarrhea can lose up to 21% of their body weight (30). Oral electrolyte administration is the main way to prevent severe dehydration induced by secretory diarrhea. These results suggest the CP may be a better option for calves with more watery diarrhea (fecal score=4) and could reduce disease duration. Nonetheless, additional studies are necessary to validate the findings and explore potential mechanisms for the effect of CP on calf diarrhea.

In addition to promoting the selection of antimicrobial resistance in enteric bacteria, many antimicrobial agents produce adverse effects on small intestinal absorption function and normal intestinal flora, which affects the growth of animals (31, 32). Therefore, growth performance is an important indicator to evaluate the efficacy of drug therapy. In the current study, the average daily gain was significantly higher in the CP group (211.45 g/d) when compared with the control group (164.56 g/d). This difference in average daily weight gain may be associated with a shorter duration of disease.

## Conclusion

This randomized clinical trial indicates that CP has a beneficial clinical effect for treatment of diarrhea in neonatal calves compared to apramycin treatment based on (1) a higher clinical cure rate, (2) a shorter duration of disease, and (3) A superior weight gain. Therefore, CP might represent a potentially effective alternative treatment option for calf diarrhea having several other advantages over antibiotic treatment.

## Data Availability Statement

The original contributions presented in the study are included in the article/Supplementary Material, further inquiries can be directed to the corresponding author/s.

## Ethics Statement

The animal study was reviewed and approved by the Institutional Animal Care and Use Committee of Lanzhou Institute of Husbandry and Pharmaceutical Sciences of the Chinese Academy of Agricultural Sciences.

## Author Contributions

SW and JZ: conceptualization. DC: methodology, investigation, and data curation. YL: formal analysis. SW: writing—original draft preparation and funding acquisition. ZY: writing—review and editing. JZ: supervision and project administration. All authors have read and agreed to the published version of the manuscript.

## Funding

This research was funded by National Natural Science Foundation of China (32172902); Key Research and Development Program of Gansu Province (20YF8NA028) and Innovation and Entrepreneurship Talent Project of Lanzhou (2018-RC-91).

## Conflict of Interest

The authors declare that the research was conducted in the absence of any commercial or financial relationships that could be construed as a potential conflict of interest.

## Publisher's Note

All claims expressed in this article are solely those of the authors and do not necessarily represent those of their affiliated organizations, or those of the publisher, the editors and the reviewers. Any product that may be evaluated in this article, or claim that may be made by its manufacturer, is not guaranteed or endorsed by the publisher.
